# Bipolar Coagulation Aneurysm Dome Remodeling (Aneurysmorrhaphy) for Middle Cerebral Artery Aneurysms

**DOI:** 10.7759/cureus.6993

**Published:** 2020-02-14

**Authors:** Charles Kulwin, Aaron Cohen-Gadol

**Affiliations:** 1 Neurosurgery, Goodman Campbell Brain and Spine, Indianapolis, USA; 2 Neurosurgery, Indiana University, Indianapolis, USA

**Keywords:** aneurysm coagulation, aneurysm remodeling, aneurysmorrhaphy, cerebral aneurysm, clip ligation, dome coagulation remodeling

## Abstract

Adequate visualization of the proximal and distal vessels and clip reconstruction of the bifurcation with complete aneurysm neck exclusion are often difficult in the setting of bulbous small- and moderate-sized middle cerebral artery (MCA) aneurysms. We describe our experience with bipolar aneurysm dome remodeling in the setting of bulbous MCA aneurysms. The charts of the senior author’s (Aaron Cohen-Gadol) patients who underwent clip ligation of their MCA aneurysm (220 patients), and more specifically those whose aneurysm clipping was facilitated through bipolar coagulation remodeling (8 patients), were reviewed. Patient demographics, aneurysm characteristics, and postoperative angiographic results were analyzed. Eight patients with eight MCA aneurysms were treated through this technique over a six-year period. Their mean age was 53 years, and six of the eight patients were women. Two involved the anterior temporal artery, and the rest were at the M1 bifurcation or trifurcation. Three of the eight were ruptured; the mean aneurysm maximum diameter was 7 mm. Postoperative angiography demonstrated complete aneurysm obliteration in seven of eight patients; one patient had an asymptomatic mild stenosis of her anterior temporal artery’s origin, and another had a small neck remnant in the setting of a highly atherosclerotic neck. On the basis of this experience, dome coagulation remodeling of small- and moderate-sized bulbous aneurysms in the setting of poor proximal and distal vessel visualization was found to be safe for facilitating aneurysm clipping and offers more desirable clip deployment.

## Introduction

Aneurysms of the middle cerebral artery (MCA) are often broad-based and incorporate the branching vessels. The projection of the aneurysm’s dome, the course and trajectory of the M2 vessels, and the accessibility of the M1 branch are all variable and require direct intraoperative identification to safely and completely clip ligate these lesions. Bulbous MCA aneurysm domes often limit adequate neck visualization and precise collapse of the aneurysm neck through clip placement. Temporary occlusion of the M1 typically relaxes the tension on the sac but may not provide adequate sac deflation to allow concise clip deployment. Trapping the aneurysm or complete regional circulatory arrest increases the risk of ischemia. To identify the relevant anatomy, temporary M1 occlusion, brain retraction, and dome manipulation can all be employed with their individual risks.

Bipolar cautery of cerebral aneurysms is a known surgical technique [1] that involves shrinking the dome after a tentative clip is placed to confirm the position of the clip blades [2-3]. This maneuver has also been used in the setting of difficult-to-clip microaneurysms, alternatively called “very small” or “blister” aneurysms [4-5]. However, in the treatment of small- and moderate-sized saccular aneurysms, dome coagulation as part of aneurysm neck exposure before clip placement has not been well documented in the peer-reviewed English literature. We have found this method very useful in facilitating visualization of the anatomy of the parent and branching vessels relative to the aneurysm neck during clip placement. We describe our technique and clinical results, and provide operative videos to demonstrate this technique.

## Technical report

**Figure 1:**
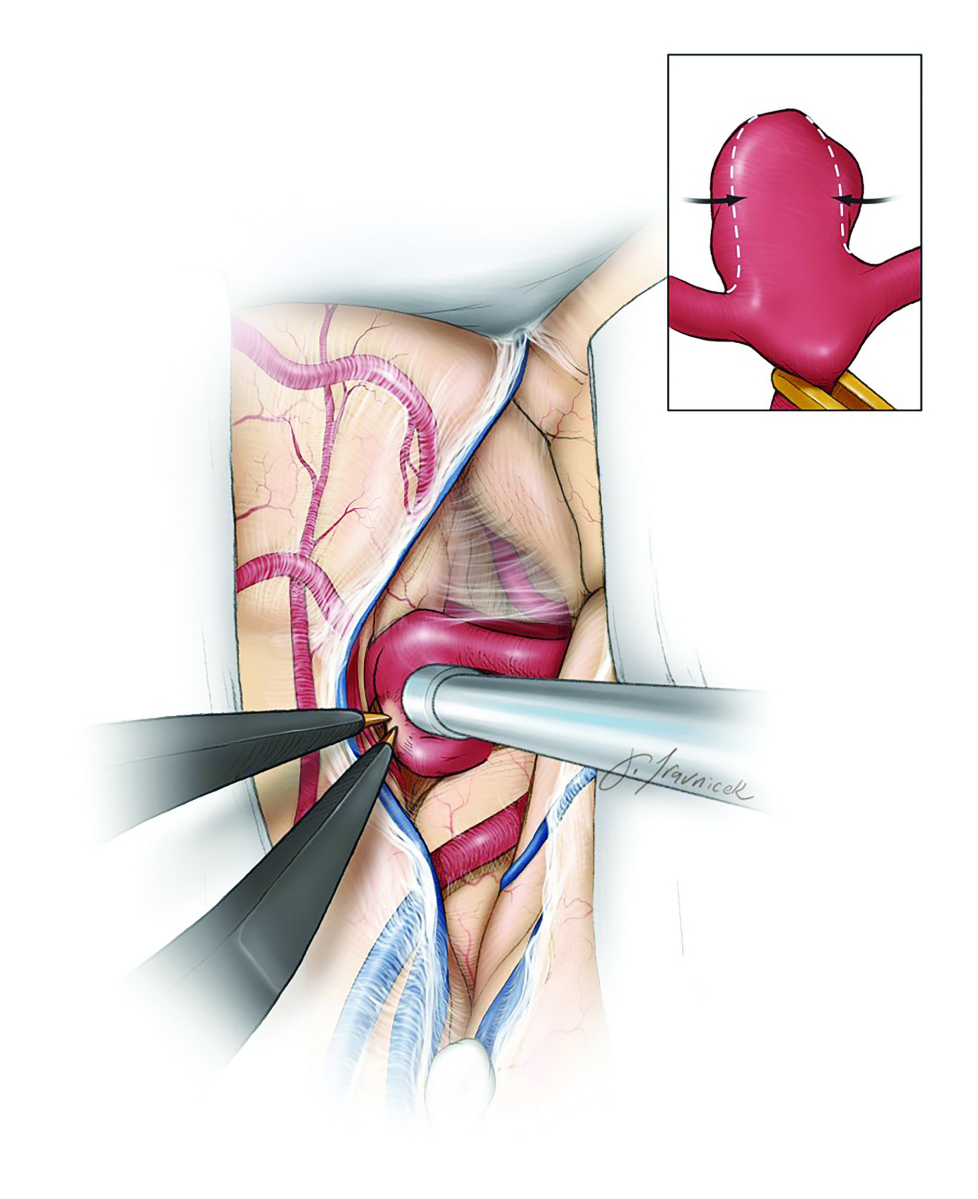
Circumferential Bipolar Coagulation Remodeling The principle of circumferential bipolar coagulation remodeling of the midbody of the aneurysm sac for its contraction so that the clip blades can be placed across the neck more effectively under direct vision. Temporary clip occlusion of the M1 is used, whereas the neck and the dome of the aneurysm (if ruptured) are spared to avoid neck injury and premature rupture, respectively. (Used with permission from The Neurosurgical Atlas by Aaron Cohen-Gadol, MD.)

After a pterional exposure of the proximal and distal Sylvian fissure, the fissure was dissected using standard microsurgical techniques, generally in a distal-to-proximal inside-to-outside fashion. In the setting of rupture, early aneurysm dome manipulation was avoided. Maximum safe dissection identifying the M1, M2s, and the anterior temporal artery (ATA) was performed. When the aneurysm dome was found to be bulbous and obstructing adequate visualization of the proximal branching arteries and the aneurysm neck, specifically the temporal M2 within our operative blind spot, the dome was evaluated for bipolar coagulation remodeling. The midsection of the dome was selected for this purpose under temporary occlusion of the M1 segment. The neck was spared, and we avoided the section of the dome with comparatively thin walls. Gentle irrigation was used continuously, whereas the midsection of the dome away from the neck and around its circumference was intermittently coagulated to gather the bulbous mass and define its neck. The atherosclerotic part of the aneurysm was also avoided (Figure 1).

We used an electrosurgical generator (Valleylab Force FX, Medtronic, Minneapolis, MN) on the bipolar setting of “10” and fine-tipped nonstick microsurgical bipolar forceps. Most of the dome must be untethered from the surrounding pia/parenchyma before its shrinkage. After dome remodeling was complete, standard clipping techniques were employed to complete clip ligation of the neck. See Videos 1 and 2 for further discussion on technical details.

**Video 1:** Broad-Base Left-Sided Complex MCA Bifurcation Aneurysm A broad-base left-sided complex MCA bifurcation aneurysm was clip ligated with the assistance of aneurysmorrhaphy. (Used with permission from The Neurosurgical Atlas by Aaron Cohen-Gadol, MD.) MCA, middle cerebral artery

**Video 2:** Right-Sided Anterior Temporal Artery Aneurysm A right-sided anterior temporal artery aneurysm was clipped through bipolar coagulation remodeling of the dome. (Used with permission from The Neurosurgical Atlas by Aaron Cohen-Gadol, MD.)

Results

Charts of the senior author’s (Aaron Cohen-Gadol) patients who had undergone microsurgical clip ligation of their MCA aneurysm were reviewed for cases of treatment through aneurysm dome bipolar remodeling. Over a six-year period, 220 patients underwent clip ligation, and 8 (4%) were identified through chart analysis and review of intraoperative surgical videos. The patients’ demographics, aneurysm characteristics, and angiographic results were collected. Their mean age was 52.9 years (range: 44-66 years); six of eight patients were women. All aneurysms involved the MCA vascular territory; half of them were on the left. Two involved the M1 ATA branch point, and the rest occurred at the M1-M2 bifurcation or trifurcation. Three were ruptured, and five were unruptured. The mean maximum aneurysm diameter was 7 mm (range: 5-8 mm). Two harbored significant atherosclerosis at the neck on intraoperative inspection.

These consecutive patients underwent aneurysmorrhaphy without any intraoperative complication. No injury to the neck or premature rupture occurred. This maneuver assisted in effective clip application while mobilizing the bulbous dome away from the working angles necessary to deploy the clip. This technique was especially helpful for closure of the distal ends of the clip blades under direct vision while preserving the proximal temporal M2 trunk usually hidden under the dome and the temporal opercula. Furthermore, simple and single clip reconstruction methodologies were adequate for aneurysm obliteration.

Radiographic outcomes were available for all patients; seven underwent detailed postoperative digital subtraction angiography using three-dimensional rotational imaging and one underwent CT angiography. Seven of eight had complete aneurysm occlusion, whereas one had a small neck remnant in the setting of an atherosclerotic neck; this remnant was expected as the clip blades tended to slide toward the M1 bifurcation intraoperatively if the blades were deployed directly over the neck. Seven of eight patients’ images demonstrated no parent or daughter vessel stenosis, whereas one patient harbored mild radiographic stenosis of the ATA with no clinical sequelae.

Case Illustration

**Figure 2:**
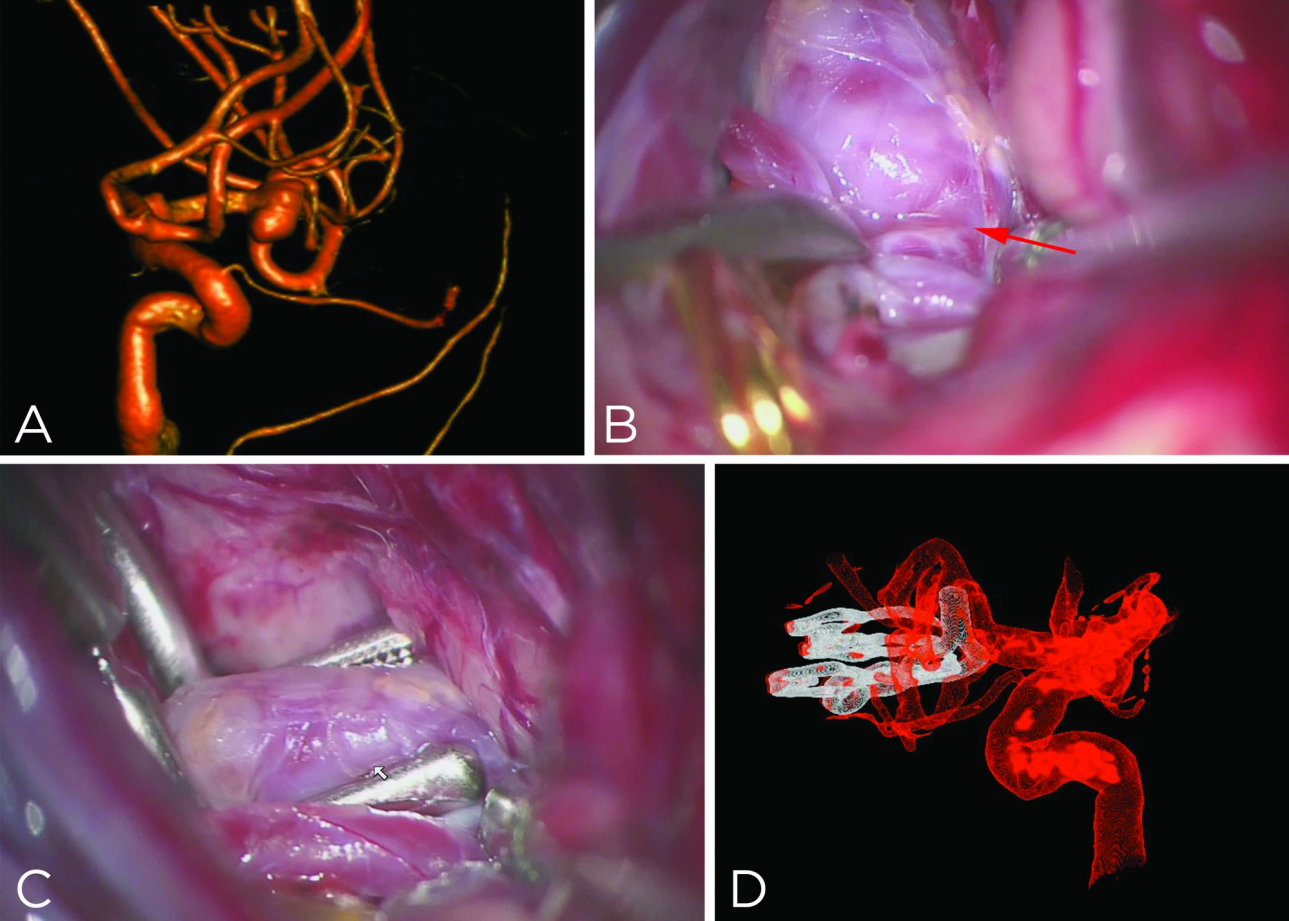
Unruptured Left-Sided Complex MCA Aneurysm (A) A 57-year-old man presented with an unruptured 7-mm left-sided complex MCA aneurysm. (B) Exposure of the aneurysm revealed a very broad base aneurysm with a minimal neck at its junction with the temporal M2 trunk (the junction is noted with the red arrow). (C) The sac was remodeled to allow for an effective application of the clip blades while maintaining the patency of the M2 trunks. Additional clips were necessary to completely exclude the sac. (D) Postoperative angiography revealed complete aneurysm obliteration. (Used with permission from The Neurosurgical Atlas by Aaron Cohen-Gadol, MD.) MCA, middle cerebral artery

A 57-year-old man presented with an unruptured 7-mm left-sided MCA aneurysm (Figure 2A) and underwent clip ligation. Exposure of the aneurysm revealed a very broad base aneurysm with a minimal neck at its junction with the temporal M2 branch (Figure 2B, the junction is noted with the red arrow). The bulbous morphology of the sac and its complex anatomy relative to the temporal M2 precluded an effective clip ligation without leaving residual aneurysm sac behind. The sac was remodeled using bipolar coagulation to allow for an effective application of the clip blades while maintaining the patency of the M2 trunks (Figure 2C). Additional clips were necessary to completely exclude the sac. Postoperative angiography revealed complete aneurysm obliteration (Figure 2D).

## Discussion

Yaşargil’s definitive microneurosurgical operative text expounds on the role of bipolar cautery in the treatment of cerebral saccular aneurysms. “In cases of a ‘complex aneurysm’, with a broad or irregular base…the aneurysm is often not amenable to initial clipping, even if the neck is adequately dissected” [6]. He enumerates multiple techniques to facilitate “perfect clip placement” including suction or forceps manipulation of the dome, temporary clip placement on parent vessels, and bipolar coagulation.

Bipolar coagulation is described “as a prelude to the initial application of the clip…as a help during the further application of the clip, and during the further manipulation of the aneurysm before final clip placement” [6]. While describing its role in controlling intraoperative rupture, Yaşargil also cautions against the hazards of aneurysm coagulation - “excessive heating…forceps-tips stick[ing] to the aneurysm…its neck may be avulsed…perforating vessels injured” - and then reinforces the importance of frequent bipolar tip cleaning, short controlled applications of cautery, proper experience with bipolar cautery on artery walls, and having several sets of forceps available [6]. Of note, bipolar coagulation will be less efficacious on calcific, atherosclerotic portions of the aneurysm complex [7], and because of the degenerative histological changes that coagulation induces, it should be avoided on the aneurysm neck at/below the site of clip placement to avoid creating a weak spot in the vessel wall that may predispose the patient to a future risk of recurrence or rupture.

The majority of Yaşargil’s operative figures, and the few literature sources describing this technique that we have been able to identify [2,3,7], describe preliminary aneurysm clipping with sequent dome coagulation before placement of a final “perfect” clip. In our experience, however, we have usually deferred preliminary/tentative clip placement because premature clip placement without adequate visualization around the neck may lead to partial clipping and sac rupture/injury to an adherent vessel. In addition, coagulation of the dome and the generation of stasis in the aneurysm sac can lead to the formation of thrombi that can embolize during the replacement of the clip blades. Finally, most of our rare aneurysms highly selected for aneurysmorrhaphy were of such complex morphology that the initial clip placement was not straightforward without placing the branching vessels at risk.

We have found aneurysm dome coagulation with temporary parent vessel occlusion to be safe in allowing complete neck dissection for proper final clip placement. Very few patients in our series underwent such a maneuver. This fact highlights our very selective philosophy in using this method because there are associated risks with coagulation of the dome, including premature rupture. Routine use of this method is not advised. Aggressive coagulation of the entire dome between the tips of the forceps was not performed. This technique should be available in the armamentarium of the surgeon during the occasional circumstances where the bulbous dome with complex morphology interferes with precise clip deployment. Atherosclerotic large or giant aneurysms are not appropriate candidates for this maneuver because the presence of the plaque will prevent sac shrinkage.

## Conclusions

We describe our experience with aneurysm dome bipolar coagulation remodeling before definitive aneurysm clipping for saccular small- and moderate-sized bulbous MCA aneurysms. In all cases, visualization of the vascular anatomy at the neck was facilitated, clip placement was effective, and no sac rupture or any other complication occurred. This preliminary experience supports the safety of this technique in select cases.
